# Effects of an Exercise for Well-Being and Physical Training Programme on Muscle Strength, Range of Movement, Respiratory Capacity and Quality of Life in Women with Fibromyalgia: A Randomized Controlled Trial

**DOI:** 10.3390/jcm12030774

**Published:** 2023-01-18

**Authors:** Juan Rodríguez-Mansilla, Abel Mejías-Gil, Elisa María Garrido-Ardila, María Jiménez-Palomares, Jesús Montanero-Fernández, María Victoria González-López-Arza

**Affiliations:** 1 ADOLOR Research Group, Department of Medical-Surgical Therapy, Medicine Faculty, Extremadura University, 06006 Badajoz, Spain; 2 Mathematics Department, Medicine Faculty, Extremadura University, 06006 Badajoz, Spain

**Keywords:** fibromyalgia, exercise for well-being, physiotherapy, muscle strength, range of movement, respiratory parameters, quality of life

## Abstract

The objective of this study was to assess the efficacy of an active exercise physiotherapy programme versus an exercise for well-being programme improving muscle strength, range of movement, respiratory capacity and quality of life of women with fibromyalgia. A randomized, assessor-blind, controlled trial was conducted. A total of 141 women diagnosed with fibromyalgia were randomized to a physiotherapy exercise group (*n* = 47), an exercise for well-being group (*n* = 47) and a control group (*n* = 47). The study lasted 4 weeks and the experimental groups received 45 min sessions performed twice a week on alternate days. The primary outcome measures were range of movement and muscle strength. The secondary outcome measures were respiratory capacity and quality of life. The results showed statistically significant improvements in the exercise for well-being and physiotherapy groups vs. the control group at week 5 in relation to joint range of movement (*p* = 0.004), muscle strength (*p* = 0.003) and quality of life (*p* = 0.002). The changes found in all the spirometry parameters seem to be associated to some of the changes in joint range of movement and muscle strength as well as quality of life. Physiotherapy and exercise for well-being improved upper limb and lower limb range of movement and the muscle strength of women with fibromyalgia.

## 1. Introduction

Fibromyalgia is a chronic rheumatic condition that can cause varying degrees of disability [1]. It is characterized by the appearance of symptoms such as pain, fatigue, musculo-skeletal disorders [1], thoracic mobility alterations [2] and respiratory disorders that have a negative impact on the quality of life of these patients [3].

Pharmacological treatment has produced meaningful improvement in 30% to 50% of patients [4]. However, a study on prevalence and impact of rheumatic diseases [5] has indicated that fibromyalgia is one of the chronic diseases associated with the highest consumption of medication.

The management of fibromyalgia can also include non-pharmacological treatments such as physiotherapy and psychological interventions, as well as socio-educational measures [6,7]. These management approaches have shown improvements on physical, psychological and social quality of life [6,7].

Exercise for well-being (Qi Gong) is also a non-pharmacological treatment approach that has been used in the last few decades [8]. The World Health Organization defines exercise for well-being as “a Traditional Chinese Medicine technique that combines movement, meditation and breathing to improve circulation, immune system and the flow of the body’s energy or Qi” [9]. Different systematic reviews and clinical trials have demonstrated that exercise for well-being improves cardiovascular system function, physical function, pain and psychological disorders associated to chronic diseases or palliative care [10,11]. Many medical academic institutions in the United States and other Western countries are seriously considering the use of exercise for well-being in medicine and psychiatry based on the positive results that different clinical trials have achieved [12,13]. However, very few studies have assessed the positive changes that exercise for well-being can bring to the management of fibromyalgia [11].

Clinical trials such as the one conducted by Burchkhard et al. [14] or the systematic review carried out by Bidonde et al. [15] suggest that physical activity or a combination of two or more types of exercises can improve respiratory capacity, pain, stiffness, fatigue and physical function. In addition, other authors affirm that aerobic exercise is one of the more effective treatment techniques for fibromyalgia based on the benefits shown in relation to sleep quality, pain relief and fatigue [16].

Physiotherapy techniques such as stretching and physical exercise, and non-conventional techniques such as exercise for well-being, are being employed in the management of chronic conditions like fibromyalgia. However, research suggests that there is a scarcity of studies that have conducted interventions to treat the respiratory disorders in fibromyalgia [3] or that compare physiotherapy and exercise for well-being to assess their effectiveness [17].

Based on all this, the objective of this clinical trial was to assess the effectiveness of physiotherapy and exercise for well-being, improving muscle strength, range of movement, respiratory capacity and quality of life in patients with fibromyalgia.

## 2. Materials and Methods

### 2.1. Design

This study was an assessor-blind randomized clinical controlled trial. Ethical approval was received from the Bioethical Commission of the University of Extremadura- Spain (Registration number: 11/2012). The study was registered in the ClinicalTrials.gov registry (NCT04328142). All the participants signed a written informed consent form. The trial was carried out and reported following the CONSORT statements and guidelines [18,19].

### 2.2. Participants

The population assessed for eligibility was women diagnosed with fibromyalgia from the Fibromyalgia Associations of Badajoz and Olivenza in Extremadura (Olivenza and Badajoz, Spain). The established inclusion criteria were: women diagnosed with fibromyalgia according to the the American College of Rheumatology Diagnostic Criteria for Fibromyalgia [20] by a specialized physician at least one year before the beginning of the study and women within an age range of 30 to 65 years old. The exclusion criteria were: to have been prescribed with physiotherapy or pharmacological treatment previous to the beginning of the study, to do regular physical exercise, to have previous knowledge or experience of exercise for well-being, to present any impairments related to mobility or deficiency of the upper or lower limb due to fibromyalgia or other pathology and pregnancy.

A total of 141 women were randomly allocated to a physiotherapy experimental group, an exercise for well-being experimental group or a control group. The randomization was carried out by an independent researcher who was not related to the trial. The researchers who were directly involved in the project had no access to any information about the randomization system or the list. An opaque bag contained 141 sealed envelopes with the group names and was kept closed during all the randomization processes except for the moment when the participant took an envelope out. At that time, the independent researcher was in charge of opening the bag and the envelope. After the first assessment, he informed each participant to which group they were allocated to. At all times and until the participants were assigned, the allocation was concealed.

The study was carried out during a total period of six weeks. This period was divided into four weeks of treatment, following the recommendations for fibromyalgia exercise programmes [21], and two weeks in which the measurements were performed. Two measurement sessions were completed: a baseline assessment which was performed at week 0 and a post-intervention assessment which was performed at week 5. The assessor was a therapist who was blinded to the allocation of the participants and had no information regarding the treatments that were applied during the treatment period. The therapists and the participants were not blinded as, due to the type of treatment applied, they were aware of the group allocation.

### 2.3. Outcome Measures

A data collection protocol was used to collect the sociodemographic data (education, working status and marital status) and the outcome measures.

The primary outcome measures were joint range of movement measured with a two-arm goniometer and muscle strength measured with the Daniels and Worthingham scale [22,23]. The secondary outcome measures were respiratory capacity (spirometry parameters) assessed with a Spirobank-G (MIR)^®^ spirometer and interpreted on the basis of a Knudson model and the literature available [24,25,26] and quality of life evaluated through the Spanish Fibromyalgia Impact Questionnaire (S-FIQ) [27,28] (Supplemental S1).

The assessment of the outcome measures was done at week 0 (pre-intervention) and at week 5 (post-intervention).

### 2.4. Interventions

The sample was allocated to three groups: two experimental groups and one control group. The experimental physiotherapy group received physiotherapy treatment, the experimental exercise for well-being group received exercise for well-being treatment, and the control group did not receive any treatment. Supplemental Table S1 presents all the relevant aspects of the intervention according to the Template for Intervention Description and Replication (TIDieR) guidelines [29].

In order to act in accordance with the beneficence and non-maleficence principles of bioethics, all the participants of the trial continued with their medical treatment during the interventions.

### 2.5. Statistical Analysis

The statistical analysis was performed by IBM SPSS 22.0 (Chicago, IL, USA). Statistical differences were considered significant at *p* < 0.05.

No previous calculation of the sample size was performed, since as many participants as possible were included in the study. Finally, between 29 and 33 participants completed the treatment in the experimental group. This sample size is considered to be enough to detect an effect size δ = 0.5 given a 2-sided level 5% t-paired test and a statistical power of 80% (as calculated with the jamovi 1.2 computer software).

The sociodemographic characteristics of the participants and the baseline values of the main outcomes were analysed and described by groups. In order to assess the homogeneity of the sample, a one-way ANOVA was carried out. The changes were analysed comparing the baseline and the final values and described by experimental groups. This was considered to be more appropriate than just analysing the post-treatment outcomes. In this main analysis and due to the big numbers of outcomes involved, a one-way multivariate analysis (Pillay’s test) was applied for each dimension with the aim of controlling type I error probability. Then, intergroup comparisons of changes were carried out by a one-way ANOVA followed by the HSD–Tukey post hoc method. Stundet’s t-paired test was also applied in order to analyze intragroup progress along the treatment.

The correlations between the age of the participants and each of the main outcomes were analyzed with Pearson’s correlation test. For each spirometry parameter, a multiple linear regression with a backward selection method was considered. The change in the spirometry parameter along the study was considered as a dependent variable. All the changes in joint range of movement and muscle strength were considered as independent variables. The objective of this analysis was to study if the changes obtained in joint range of movement or muscle strength could partially explain the changes observed in spirometry parameters. In the same way, a multiple linear regression was also applied. The changes in the Spanish Fibromyalgia Impact Questionnaire were considered as a dependent variable and all the changes in spirometry were considered as independent ones. The *t*-test was applied to other comparisons that involved just two mean values.

## 3. Results

**Figure 1:**
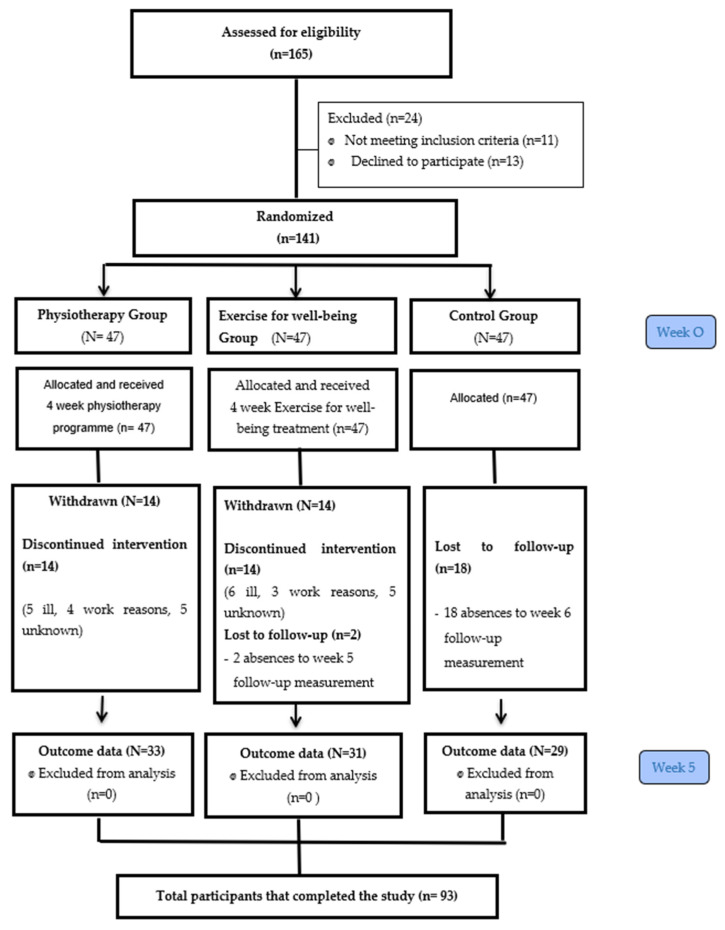
CONSORT flow diagram.

A total of 93 women finished the intervention and completed all measurements of the study. The reasons for the drop out can be found in Figure 1 which presents the flow diagram according to the CONSORT guidelines. The physiotherapy group had 33 participants, the exercise for well-being group had 31 and the control group had 29. There were 48 losses in total during the treatment period and the follow-up and therefore, their data were excluded from the statistical analysis.

Table 1 provides all the figures related to the sociodemographic data collected. The mean age of the sample was 52.24 ± 6.19 and the age range was 34 to 65 years old. As expected, the age showed a significant correlation with many baseline outcomes (muscle strength of cervical spine extensors, cervical spine flexors, shoulder adductors, shoulder horizontal flexors, hip flexors, hip extensors, hip adductors and hip abductors, range of movement of shoulder extensors, forced vital capacity (FVC) and forced expiratory volume in 1 s (FEV1)). The results indicate that these outcome measures became worse with age (see Table 2). Nevertheless, significant correlations between the age and the changes experienced during the study were scarcely detected (except for hip extensor and abductors muscle strength which improved with age).

Baseline data have been summarized and detailed by experimental groups in Table 3. The results of one-way ANOVA showed that there were no significant differences between the experimental groups, with the only exception of the shoulder extensor range of movement and shoulder adductors’ muscle strength. This can be explained by the assumption of 5% in significance level.

The changes of all the primary and secondary outcome measures (i.e., the difference between the post-treatment and the pre-treatment measurements) are summarized in Table 3. The results are also presented by groups and dimensions. According to the t-paired test, the control group did not show any changes (except for muscle strength of the cervical spine flexors, hip flexion range of movement and force expiratory flow (FEF) 25–75%). However, both the physiotherapy and exercise for well-being groups achieved significant improvements in most outcome measures (underlined in Table 4).

Nevertheless, the results also indicate that the control group had a tendency to show a slight improvement in many outcome measures. However, these changes were not statistically significant. In order to analyse this effect, it was necessary to study when the improvements of physiotherapy and exercise for well-being groups were significantly better compared with those showed by the control group. This analysis was made firstly for each dimension by a one-way multivariate ANOVA. Significant results were obtained for muscle strength (*p* = 0.003), for joint ROM (*p* = 0.004) and for spirometry (*p* = 0.039) as well as for the single dimension quality of life (*p* = 0.002). This indicates that there are differences in these dimensions in general. Then, each outcome was analysed separately throughout a one-way ANOVA and Tukey’s HSD post hoc method. Cases of improvement with respect to the control group are marked in bold in Table 4. Therefore, the physiotherapy and the exercise for well-being groups performed much better than the control group in most of the primary outcome measures, except for spirometry parameters.

When comparing the physiotherapy group with the exercise for well-being group, the results show better performance in the first one, at least in sample terms. These results can be seen in Table 4. However, significant differences between both therapies were barely found (only for the variables hip adductors muscle strength and FEF 25–75%).

In relation to the secondary outcome measures, as was pointed out firstly, quality of life showed better performance assessed with the S-FIQ in the physiotherapy group and the exercise for well-being group (*p* = 0.002). However, there were no significant differences between both groups.

Besides, we analysed the relationship between secondary and primary outcomes. A multiple linear regression with backward selection method was considered for every spirometry parameter. The change in the spirometry parameter was considered as a dependent variable and all the changes in range of movement and muscle strength were considered as explanatory variables. The results obtained are shown in Table 5 and indicate that, for every spirometry parameter, changes along the study could be associated to some of the changes in range of movement and muscle strength, especially for peak expiratory flow (PEF) (R = 0.467, *p* = 0.001) and forced expiratory flow (FEF) 25–75% (R = 0.450, *p* = 0.001). The outcomes selected by the backward method belonged to the range of movement type in the case of forced expiratory volume in 1 second (FEV1) y FEV1%, to the muscle strength in the case of forced expiratory time (FET) and to both range of movement and muscle strength in the case of forced vital capacity (FVC), peak expiratory flow (PEF) and forced expiratory flow 25–75%.

In the same way, we considered a multiple linear regression model to explain the changes found in relation to the Spanish Fibromyalgia Impact Questionnaire from changes in the spirometry. The results obtained were R = 0.311 and *p* = 0.010. The backward method chose FEV1 and FEF 25–75% which could suggest that the improvements in these outcome measures are related to the improvements in the Spanish Fibromyalgia Impact Questionnaire.

## 4. Discussion

The results of this study indicate that the exercise for well-being and the physiotherapy programmes had better results in comparison to the control group in the improvement of joint range of movement, muscle strength and spirometry values, therefore favourably improving the patients’ quality of life.

We have observed the benefits that physiotherapy and exercise for well-being produce on range of movement and muscle strength in all the joints. Few studies carried out in adults with fibromyalgia relate these two disciplines and analyse their effect on those variables [17,30]. The results obtained by Stephens et al. [30] coincide with ours, as they also found a significant improvement which was superior in the group that performed active exercise. We consider that the improvements obtained by the physiotherapy group could mainly have been due to the use of active kinesiotherapy. Exercise for well-being employs a foundation of stretching exercises, while kinesiotherapy is dynamic in practice, and requires more muscular involvement in a short period of time, therefore improving resistance and muscular strength. Despite these differences in their practice, there were no clear differences in relation to the results in both groups.

There is evidence in the literature that also indicates an improvement in range of movement and muscle strength after completing physiotherapy treatment. However, unlike our study, the research available has only analysed or obtained results in some lower limb muscles. The study carried out by Tomas-Carus et al. [31] is an example. The authors concluded that aquatic exercise improves the strength of knee flexors and knee concentric and eccentric extensors of women with fibromyalgia. Other examples are the studies conducted by Jones et al. [32] and Tran et al. [33]. The study by Jones et al. [3] showed how 68 women with fibromyalgia improved their general activity levels after completing an adapted muscular strengthening programme. The research by Tran et al. [33] showed that after physiotherapy treatment on patients with fibromyalgia, there was a significant improvement in the hip abductors, the walking biomechanics and functional performance in general.

People with fibromyalgia can present an obstructive spirometric pattern [34] and alterations in respiratory values. This can be due to complications or weaknesses in the musculature that involves breathing, causing a reduction in thoracic mobility and, in particular, alterations in FEV1 and FVC. This, at the same time, has a negative impact on the quality of life of these patients [2,35].

Some authors have related some breathing conditions, such as obstructive respiratory patterns, with upper and lower limb dysfunction [36,37,38]. These impairments can be caused by atrophy or decreased muscle strength [39] and have a negative effect on functional capacity and quality of life [40]. Based on this, we consider that the range of movement and muscle strength improvements obtained in our study could have influenced the improvement of the spirometry values. The active exercise programme focused on the upper and lower limb. At the same time, the stretching exercises and thoracic expansion exercises performed with exercise for well-being achieved muscle strengthening. Muscle strengthening has been achieved not only on the muscles directly involved in breathing (Diaphragm, Pectorals, Serratus Anterior, Latissimus Dorsi or abdominal muscles) but also, in general, in the upper and lower limb muscles.

The results of our study showed improvements in PEF and FET in the exercise for well-being group and in FEV1, FEV 1%, FET and FEF 25–75% in the physiotherapy group. This indicates that there has been an improvement on the obstructive parameters, which means a positive change on the air expiration speed and therefore an improvement in respiratory mechanics and muscle work. No studies that have compared physiotherapy and exercise for well-being and analysed their effect on respiratory parameters have been found in the literature. Moreover, scarce studies on fibromyalgia and respiratory function have been published and those that are available give contradictory results [3].

Our results coincide with those from Chan et al. [41] and Tong et al. [42] that support the fact that Tai Chi exercise for well-being can improve respiratory function, FEV1, and activity tolerance levels of patients with chronic obstructive pulmonary disease (COPD).

The quality of life of the participants in our study improved significantly, as can be interpreted from the results of the Spanish Fibromyalgia Impact Questionnaire. Both study groups showed positive changes in this outcome measure compared with the control group. These results coincide with numerous studies which conclude that physical activity, exercise programmes, or exercise for well-being improve fibromyalgia symptoms and have a positive impact on functional capacity and quality of life [11,43].

### Study Limitations

The difficulty of learning the exercises for well-being was a limitation of our study. That is why, previous to the performance of exercises, explanations of key aspects such as control of breathing, mind and body posture were necessary. We considered that 4 weeks of intervention could not be enough time to obtain all the expected results, although there are studies in the literature with the same period of interventions as ours [44]. However, increasing the intervention period could have had a negative effect on our initial concern: treatment adherence. Our previous experience working with fibromyalgia patients [45] and the study conducted by Bosch et al. [46] had losses in participation or follow-up that coincide with our results.

The outcome measures analysed in this study were muscle strength, joint ROM, respiratory capacity and quality of life as the objective of this trial was to assess the effectiveness of physiotherapy and exercise for well-being improving those symptoms in patients with fibromyalgia. Regarding quality of life, although the S-FIQ includes items related to important fibromyalgia symptoms such as fatigue and pain, only the total score was analysed. Therefore, we consider that it would be interesting to analyse the items of this scale related to important fibromyalgia symptoms separately in further research.

Structured questionnaires were used and administrated only by one researcher who was independent to the study in order to avoid information bias. We also consider that having a bigger sample will allow to assess the statistical differences between the two experimental groups, as in our study and based on our data, a statistical difference was hardly found. In addition, a longer period of follow-up would have probably given a better overview of the effects of the treatments.

## 5. Conclusions

Physiotherapy and exercise for well-being improve upper limb and lower limb range of movement and muscle strength of women with fibromyalgia. This leads to a positive change on spirometry parameters, which likewise have a positive impact on quality of life.

## Figures and Tables

**Table 1:** Socio-demographic characteristics of the participants.

		Mean ± S.D.	N
Age		52.24 ± 6.19	
VAS		7.47 ± 1.75	
Gender	Male		0
	Female		93
Working status	Housewife		41
	Unemployed		10
	Employed		23
	Unable to work		18
	Retired		1
Marital status	Married		81
	Lives with her partner		2
	Single		2
	Separated		2
	Divorced		3
	Widow		3
Education	With no studies		11
	Primary Education		40
	Secondary Education		23
	Bachelor’s Degree		19
Smoker	No		25
	Yes		68

VAS: visual analogue Scale.

**Table 2:** Correlation between age and baseline outcomes and between age and changes.

Outcome Measures	Pearson’s R	
	Baseline	Change from Baseline
**muscle strength**		
Cervical spine flexors	−0.207 *	0.113
Cervical spine extensors	−0.263 *	0.173
Shoulder flexors	−0.137	0.157
Shoulder extensors	−0.175	0.170
Shoulder abductors	−0.225 *	0.134
Shoulder horizontal flexors	−0.258 *	0.192
Hip flexors	−0.269 **	0.191
Hip extensors	−0.344 **	0.271 **
Hip abductors	−0.421 **	0.277 **
Hip adductors	−0.295 **	0.165
**joint rom**		
R Shoulder flexion	0.072	0.017
R Shoulder extension	−0.249 *	0.092
R Shoulder abduction	−0.140	0.109
R Shoulder external rotation	−0.143	0.104
R Shoulder internal rotation	−0.147	0.100
L Shoulder flexion	−0.073	0.029
L Shoulder extension	−0.014	−0.044
L Shoulder abduction	−0.092	−0.017
L Shoulder internal rotation	−0.116	0.109
L Shoulder external rotation	−0.087	−0.124
R hip flexion	0.100	−0.126
R hip extension	−0.033	0.079
R hip abduction	0.022	−0.132
L hip flexion	0.016	−0.148
L hip extension	0.057	−0.066
L hip abduction	−0.014	0.047
**S-FIQ**	0.115	0.0221
**spirometry**		
FVC	−0.225 *	−0.005
FEV1	−0.214 *	−0.037
FEV1%	−0.006	0.036
PEF	−0.030	0.091
FEF 25–75%	−0.130	0.038
FET	0.083	−0.003

**Note**: * *p* < 0.05, ** *p* < 0.01. ROM: Range of movement; R: right; L: left; S-FIQ: Spanish Fibromyalgia Impact Questionnaire; FVC: forced vital capacity; FEV1: forced expiratory volume in 1 s; FEV1%: FEV1/FVC ratio; PEF: peak expiratory flow; FEF 25–75%: forced expiratory flow at 25 to 75% interval; FET: forced expiratory time; EWBG: exercise for well-being group; CG: control group; FG: Physiotherapy group.

**Table 3:** Baseline data with between-groups comparison.

Baseline Outcome Measures	Mean ± SD			Anova *p*-Value
	CG (N = 29)	FG (N = 33)	EWBG (N = 31)	
**muscle strength**				
Cervical spine flexors	4.10 ± 0.72	4.03 ± 0.73	3.87 ± 0.76	0.459
Cervical spine extensors	4.17 ± 0.76	3.97 ± 0.85	3.87 ± 0.76	0.331
Shoulder flexors	4.28 ± 0.80	3.91 ± 0.77	3.94 ± 0.89	0.160
Shoulder extensors	4.14 ± 0.79	3.73 ± 0.88	4.00 ± 0.82	0.143
Shoulder abductors	4.10 ± 0.77	3.79 ± 0.78	4.06 ± 0.85	0.238
Shoulder horizontal flexors	4.28 ± 0.59	3.76 ± 0.94	4.16 ± 0.86	0.034
Hip flexors	4.24 ± 0.74	3.94 ± 0.83	3.94 ± 0.77	0.227
Hip extensors	4.00 ± 0.93	3.85 ± 0.83	3.94 ± 0.85	0.789
Hip abductors	4.21 ± 0.68	3.97 ± 0.68	4.13 ± 0.85	0.436
Hip adductors	4.14 ± 0.74	3.88 ± 0.82	4.13 ± 0.88	0.362
**joint rom**				
R Shoulder flexion	131.83 ± 25.16	124.39 ± 22.12	131.48 ± 22.43	0.357
R Shoulder extension	42.31 ± 10.50	46.12 ± 10.32	50.81 ± 10.40	0.008
R Shoulder abduction	117.31 ± 23.21	106.97 ± 20.46	116.84 ± 25.99	0.140
R Shoulder external rotation	68.83 ± 19.35	69.06 ± 16.60	76.90 ± 10.78	0.081
R Shoulder internal rotation	77.07 ± 16.21	74.39 ± 15.36	79.94 ± 9.50	0.290
L Shoulder flexion	131.14 ± 27.88	131.12 ± 29.31	127.73 ± 23.89	0.854
L Shoulder extension	42.45 ± 10.44	48.79 ± 9.71	50.90 ± 12.27	0.010
L Shoulder abduction	117.41 ± 28.14	115.85 ± 24.40	116.50 ± 25.88	0.973
L Shoulder internal rotation	74.48 ± 18.85	74.27 ± 16.38	81.37 ± 9.17	0.128
L Shoulder external rotation	66.52 ± 17.33	68.73 ± 15.25	69.93 ± 14.95	0.074
R hip flexion	52.38 ± 16.83	50.61 ± 16.81	54.94 ± 17.69	0.599
R hip extension	18.41 ± 9.44	17.00 ± 7.38	20.06 ± 7.43	0.322
R hip abduction	35.72 ± 11.45	37.06 ± 14.64	38.29 ± 11.72	0.739
L hip flexion	54.69 ± 19.50	54.64 ± 18.24	59.26 ± 16.61	0.517
L hip extension	17.48 ± 7.62	16.94 ± 6.52	17.74 ± 7.00	0.897
L hip abduction	41.21 ± 11.87	39.33 ± 11.17	42.29 ± 13.14	0.613
**S-FIQ**	68.86 ± 13.34	67.21 ± 16.51	65.35 ± 14.95	0.667
**spirometry**				
FVC	2.93 ± 0.65	2.76 ± 0.50	2.87 ± 0.67	0.522
FEV1	2.45 ± 0.63	2.30 ± 0.73	2.48 ± 0.57	0.499
FEV1%	83.31 ± 12.31	83.09 ± 20.38	83.87 ± 11.79	0.979
PEF	3.72 ± 1.13	5.55 ± 11.12	4.16 ± 1.51	0.534
FEF 25–75%	2.76 ± 0.74	2.76 ± 1.03	2.77 ± 0.84	0.997
FET	3.00 ± 2.24	3.79 ± 3.54	3.87 ± 2.72	0.446

**Note**: ROM: Range of movement; R: right; L: left; S-FIQ: Spanish Fibromyalgia Impact Questionnaire; FVC: Forced vital capacity; FEV1: forced expiratory volume in 1 second; FEV1%: FEV1/FVC ratio; PEF: peak expiratory flow; FEF 25–75%: forced expiratory flow at 25 to 75% interval; FET: forced expiratory time; EWBG: exercise for well-being group; CG: control group; FG: Physiotherapy group.

**Table 4:** Changes of the outcome measures analysed with the t-paired test within groups and with Anova and Tukey’s HSD between groups.

Evolution in Outcomes Post-Pre Intervention	Mean ± SD by Group, Cohen’s d of *t*-Test * Intra-Group			Inter-Group Anova # *p*-Value
**primary outcome measures**				
	CGl (N = 29)	FG (N = 33)	EWBG (N = 31)	
**muscle strength**				
Cervical spine flexors	0.28_a_ * ± 0.65, d = 0.425	0.58_a_ ** ± 0.66, d = 0.869	0.61_a_ ** ± 0.72, d = 0.857	0.114
Cervical spine extensors	0.28_a_ ± 0.80, d = 0.346	**0.82_b_** ** ± 0.81, d = 1.012	**0.81_b_** ** ± 0.65, d = 1.233	0.009
Shoulder flexors	0.31_a_ ± 0.97, d = 0.321	**0.85_b_** ** ± 0.76, d = 1.124	0.81_a,b_ ** ± 0.79, d = 1.018	0.025
Shoulder extensors	0.34_a_ ± 1.08, d = 0.320	**1.18_b_** ** ± 0.88, d = 1.340	0.65_a,b_ ** ± 0.80, d = 0.809	0.002
Shoulder abductors	0.28_a_ ± 1.00,d= 0.277	**0.91_b_** ** ± 0.80, d = 1.130	0.65_a,b_ ** ± 0.80, d = 0.809	0.019
Shoulder horizontal flexors	0.10_a_ ± 0.90, d = 0.115	**0.97_b_** ** ± 0.95, d = 1.019	0.61_a,b_ ** ± 0.76, d = 0.806	0.001
Hip flexors	0.03_a_ ± 0.82, d = 0.042	**0.82_b_** ** ± 0.81, d = 1.012	**0.77_b_** ** ± 0.72, d = 1.080	0.000
Hip extensors	0.38_a_ ± 1.21, d = 0.314	0.91_a_ ** ± 0.88, d = 1.034	0.84_a_ ** ± 0.86, d = 0.975	0.083
Hip abductors	0.14_a_ ± 0.58, d = 0.237	**0.82_b_** ** ± 0.73, d = 1.126	0.48_a,b_ ** ± 0.68, d = 0.715	0.001
Hip adductors	0.17_a_ ± 0.47, d = 0.368	**0.91_b_** ** ± 0.88, d = 1.034	0.42_a_ ** ± 0.67, d = 0.624	0.000
**joint rom**				
R Shoulder flexion	6.69_a_ ± 20.65, d = 0.324	25.21_b_ ** ± 20.07, d = 1.256	18.45_b_ ** ± 17.37, d = 1.062	0.001
R Shoulder extension	2.45_a_ ± 8.79, d = 0.279	4.42_a_ * ± 10.19, d = 0.434	4.45_a_ ** ± 8.91, d = 0.500	0.638
R Shoulder abduction	7.79_a_ ± 27.60, d = 0.282	**40.36_b_** ** ± 29.50, d = 1.368	**30.97_b_** ** ± 28.14, d = 1.100	0.000
R Shoulder external rotation	4.62_a_ ± 18.54, d = 0.249	11.15_a_ ** ± 15.78, d = 0.707	6.87_a_ ** ± 8.74, d = 0.786	0.216
R Shoulder internal rotation	−1.55_a_ ± 17.20, d = −0.090	**9.33_b_** ** ± 9.79, d = 0.953	4.77_a,b_ ** ± 9.81, d= 0.487	0.004
L Shoulder flexion	9.45_a_ ± 26.44, d = 0.357	15.27_a_ ** ± 23.37, d = 0.654	24.13_a_ ** ± 22.80, d = 1.058	0.068
L Shoulder extension	2.83_a_ ± 9.93, d = 0.285	4.97_a_ ** ± 7.62, d = 0.652	5.37_a_ * ± 10.62, d = 0.505	0.539
L Shoulder abduction	8.79_a_ ± 27.98, d = 0.314	**29.88_b_** ** ± 28.19, d = 1.060	**33.60_b_** ** ± 30.88, d = 1.008	0.003
L Shoulder internal rotation	4.14_a_ ± 16.78,d= 0.247	8.00_a_ ** ± 10.86, d = 0.736	4.37_a_ ** ± 7.31, d = 0.597	0.371
L Shoulder external rotation	−0.07_a_ ± 19.55, d = −0.004	**12.42_b_** ** ± 12.96, d = 0.959	**9.53_b_** ** ± 11.44, d = 0.834	0.004
R hip flexion	3.28_a_ ± 14.68, d = 0.223	**12.82_b_** ** ± 13.52, d = 0.948	12.26_a,b_ * ± 17.78, d = 0.689	0.031
R hip extension	1.03_a_ ± 6.39, d = 0.162	**7.24_b_** ** ± 6.58, d = 1.100	4.39_a,b_ ** ± 5.23, d = 0.838	0.001
R hip abduction	−2.66_a_ ± 8.68, d = −0.306	**6.94_b_** ** ± 16.59, d = 0.418	**6.13_b_** ** ± 7.99, d = 0.767	0.004
L hip flexion	7.79_a_ ** ± 13.87, d = 0.562	15.76_a_ ** ± 14.30, d = 1.102	11.55_a_ ** ± 16.00, d = 0.722	0.110
L hip extension	0.86_a_ ± 5.53, d = 0.156	**6.15_b_** ** ± 6.90, d = 0.891	**4.81_b_** ** ± 4.98, d = 0.966	0.002
L hip abduction	1.48_a_ ± 11.41, d = 0.130	**10.61_b_** ** ± 11.81, d = 0.8981	5.87_a,b_ ** ± 7.54, d = 0.779	0.004
**secondary outcome measures**				
	CGl (N = 29)	FG (N = 33)	EWBG (N = 31)	
**S-FIQ**	0.59_a_ ± 7.10, d = 0.083	**−9.42_b_** ** ± 13.07, d = −0.721	**−7.65_b_** **± 12.55, d = −0.6092	0.002
**spirometry**				
FVC	−0.10a ± 0.49, d = −0.212	0.12a ± 0.70, d = 0.174	−0.06a ± 0.57, d = 0.112	0.283
FEV1	−0.03_a_ ± 0.68, d = −0.051	0.33_a_ *± 0.82, d = 0.408	0.06_a_ ± 0.57, d = 0.112	0.104
FEV1%	0.03_a_ ± 11.79, d = 0.003	7.85_a_ * ± 20.69, d = 0.379	0.29_a_ ± 13.75, d = 0.021	0.094
PEF	0.07_a_ ± 1.03, d = 0.067	0.85_a_ ± 2.61, d = 0.325	0.55_a_ ** ± 0.96, d = 0.571	0.219
FEF 25–75%	−0.24_a_ ± 0.58, d = −0.419	**0.79_b_** ** ± 1.34, d = 0.588	0.19_a_ ± 0.79, d = 0.244	0.001
FET	0.59_a_ ± 4.28, d = 0.137	2.45_a_ ** ± 3.01, d = 0.815	1.35_a_ ** ± 1.78, d = 0.761	0.069

**Note 1**: ROM: Range of movement; R: right; L: left; S-FIQ: Spanish Fibromyalgia Impact Questionnaire; FVC: forced vital capacity; FEV1: forced expiratory volume in 1 second; FEV1%: FEV1/FVC ratio; PEF: peak expiratory flow; FEF 25–75%: forced expiratory flow at 25 to 75% interval; FET: forced expiratory time; EWBG: exercise for well-being group; CG: control group; FG: physiotherapy group. **Note 2:** The symbols * and ** mean significant intra-group improvement (*p* < 0.05 and *p* < 0.01, respectively) according to the t-paired test within group. # Averages in **bold** mean improvements in the Physiotherapy group or exercise for well-being group significantly different from the improvements in the control group, according to Tukey’s post hoc method. It happens when the subscript of the experimental group is just b, different from the control group’s one which is a.

**Table 5:** Changes in spirometry explained by the differences in muscle strength and range of movement according to multiple linear regression.

Spirometry	Multiple Regression R	*p*-Value	Type *	
FVC	0.372	0.034	ROM	MS
FEV1	0.356	0.002	ROM	
FEV1%	0.376	0.004	ROM	
PEF	0.467	0.001	ROM	MS
FEF 25–75%	0.450	0.001	ROM	MS
FET	0.332	0.008	MS	

**Note**: ROM: Range of movement; MS: Muscle strength; FVC: Forced vital capacity; FEV1: forced expiratory volume in 1 s; FEV1%: FEV1/FVC ratio; PEF: peak expiratory flow; FEF 25–75%: forced expiratory flow at 25 to 75% interval; FET: forced expiratory time. * Type of remaining outcomes after backward selection.

## Data Availability

The data underlying this article cannot be shared publicly to maintain the privacy of individuals that participated in the study. The data will be shared on reasonable request to the corresponding author.

## Supplementary Materials

The following are available online at https://www.mdpi.com/article/10.3390/jcm12030774/s1, Supplemental S1: Measurement tools and procedures. Table S1: Descriptions of the interventions conducted following the Template for Intervention Description and Replication (TIDieR).
