# Supplementary material for: Effects of an Exercise for Well-Being and Physical Training Programme on Muscle Strength, Range of Movement, Respiratory Capacity and Quality of Life in Women with Fibromyalgia: A Randomized Controlled Trial
Source: J Clin Med. 2023 Jan 18;12(3):774. doi: 10.3390/jcm12030774 (PMC9918138; doi:10.3390/jcm12030774)
Supplement: Supplementary file 1 [file jcm-12-00774-s001.zip › jcm-2124915-supplementary.pdf]

Journal of
Clinical Medicine

MDPI

SUPPLEMETARY MATERIAL

Supplemental S1. Measurement tools and procedures

The outcome measures were assessed with the following measurement tools:

Muscle strength. The Daniels and Worthingham scale (1942) was used to assess this variable. This is the modified version of the scale created by Lovett in 1912 [1,2]. Strength was tested at the shoulder and hip muscles. The shoulder flexors, extensor, adductors and abductors as well as the hip flexors were assessed with the participant in sitting position. The hip extensors were assessed in prone position while the hip adductors and abductors were tested in supine on a plinth. The assessment started on grade 3 where the muscle can perform a complete movement against gravity. Posteriorly the test was continued in a higher or lower grade according to the response of the participant.

Joint range of movement. A two-arm goniometer was used for the assessment of the active range of movement throughout the course of the study The range of movement of both shoulders (flexion, extension, abduction, external and internal rotation) and hips (flexion, extension and abduction) were evaluated. Joint range of movement was measured with the patient sitting except for the shoulder rotations and the hip movements that were assessed in supine. Each movement was measured three times with a 10 second rest in between tests. The best score of the three attempts was recorded.

Spirometry parameters. The spirometry was completed with a Spirobank-G (MIR)® spirometer. The results of the test were interpreted with the software Winspiro 1.3. The parameters measured were: forced vital capacity (FVC), forced expiratory volume in 1 second (FEV1), FEV1/FVC ratio (FEV1%), forced expiratory flow at 25 to 75% interval (FEF 25-75%), peak expiratory flow (PEF) and forced expiratory time (FET). The reference values were based on the Knudson model and the literature available[3-5]. For a 50 year old woman with 1,65 cm of height and 65 kg of weight, the reference values are: FVC= 3.611; FEV1= 2.7505; FEV1%= 76.17%; FEF 25-75%= 2.625 and PEF= 6.222.

The spirometry procedure was carried out in the sitting position. After a detailed explanation of the test, the participant was given a cardboard mouthpiece which was connected to the spirometry machine. A clip was placed on the nose previous to the test. Then she was asked to place her lips tightly around the mouthpiece and perform a maximum inspiration followed by a maximum expiration. The expiration had to be continuous, with a constant flow and with a minimum duration of 6 seconds. Testing was repeated three times by each participant with a resting time of one minute between tests. The best score was registered for the study.

Quality of life. The impact of the condition on the patient’s quality of life was assessed with the Spanish Fibromyalgia Impact Questionnaire (S-FIQ)[6]. This is the Spanish adaptation of the Fibromyalgia Impact Questionnaire[7]. The S-FIQ has a reliability coefficient of .81. The maximum score is 100 and the higher the result obtained, the higher the impact of the condition on the person.

Table S1. Descriptions of the interventions conducted following the Template for Intervention Description and Replication (TIDieR)[8].

Item nº	Item<br>Physiotherapy group
1.	**BRIEF NAME**<br>Physiotherapy intervention: active exercise programme
2.	**WHY**<br>The exercise programme aimed to work on all the musculoskeletal system.
3.	**WHAT**<br>Materials: Exercise mats
4.	Procedures: The exercise programme included a warm up of 3 to 5 minutes walking, active mobilisation exercises of the shoulders, spine and hips, static balance exercises and stretches. The shoulder, hip and cervical spine exercises were performed in standing. The thoracic spine and lumbar spine were done on an exercise mat. All exercises were performed in coordination with controlled gentle breathing. Each mobilisation exercise was done at maximum range of movement, was maintained for 10 seconds and repeated 6 times with eyes open and closed. All movements were done slowly and avoiding pain and fatigue.
5.	**WHO PROVIDED**<br>A qualified physiotherapist provided the treatment. He was a member of the Spanish Chartered Society of Physiotherapists and was trained in exercise for Fibromyalgia
6.	**HOW**<br>All the sessions were provided face-to-face.
7.	**WHERE**<br>The location were the treatment sessions were carried out were the Physiotherapy laboratory classrooms of the University.
8.	**WHEN and HOW MUCH**<br>The physiotherapy sessions lasted for 45 minutes and were done twice a week (Tuesdays and Thursdays). The treatment was conducted during 4 weeks.
9.	**TAILORING**<br>Not applicable
10.	**MODIFICATIONS**<br>Not applicable
11.	**HOW WELL**<br>Intervention adherence and attendance at treatment sessions was recorded by the professional guiding the treatment.

Item nº	Item<br>QI Gong group
1.	**BRIEF NAME**<br>Qi gong exercises
2.	**WHY**<br>The Qi Gong exercises used are based on centennial therapeutic exercises from Daoyin, Wiqinxi, Yijinjing and Baduanjin which are transmitted orally from master to disciple. The figures combine mental concentration and abdominal breathing during the performance of balance, flexibility and coordination body movements.
3.	**WHAT**<br>Materials: No materials required

**4.**	Procedures: The exercises performed during the sessions were the 'twenty Wang Ziping figures for health and longevity'.<br>According to the Chinesse Traditional Medicine, the names of the exercises were: exercise 1: Breath out the bad air and breath in fresh air; exercise 2: the children receive food; exercise 3: the phoenix tightens its grip; exercise 4: Tension the arc in two directions; exercise 5: The king raises the tripod; exercise 6: Raise your arms to pull the fruit out; exercise 7: Look for the bottom of the sea; exercise 8:The rhinoceros looks at the moon; exercise 9: Lotus leaves swing in the wind; exercise 10: Push a stone tablet; exercise 11: Introduce a hand in Mount Huashan; exercise 12: Put aside the mane of a white horse; exercise 13: The phoenix spreads its wings; exercise 14: The carpenter handles a drill; exercise 15: The black dragon turns around; exercise 16: The Arat dominates a tiger; exercise 17: The white crane circles its knees; exercise 18: The friar squats; exercise 19: Kick with the lower half of the legs; exercise 20: The fairy walks back and forth.<br>Each figure was repeated 6 times.
**5.**	**WHO PROVIDED**<br>The Qi Gong was guided by a Qi Gong teacher with 20 years of experience and qualified by the Qi Gong International Institute (funded by Yves Requena).
**6.**	**HOW**<br>All the sessions were provided face-to-face.
**7.**	**WHERE**<br>The treatment sessions were carried out in the outside gardens of the Faculty of Medicine of the University of Extremadura.
**8.**	**WHEN and HOW MUCH**<br>The Qi Gong sessions lasted for 45 minutes and were done twice a week (Mondays and Wednesdays). The treatment was conducted during 4 weeks..
**9.**	**TAILORING**<br>Not applicable
**10.**	**MODIFICATIONS**<br>Not applicable
**11.**	**HOW WELL**<br>Intervention adherence and attendance at treatment sessions was recorded by the professional guiding the treatment.

References

1. Igual C, Muñoz E, Aramburu C. Fisioterapia General. Cinesiterapia (General physiotherapy. Kinesiotherapy). Madrid: Síntesis; **1996**.
2. Talavera A, Almazán G. Fisioterapia: La Salud en buenas manos (Physiotherapy: Health in good hands). Madrid: Instituto Alcalá de Ciencias y Especialidades de la Salud (Alcalá Institute of Health Sciences and Specialities) **2000.**
3. Falaschetti E, J Laiho, Primatesta P, et al. Las ecuaciones de predicción de la función pulmonar normal y baja de la Encuesta de Salud de Inglaterra (Normal and low lung function prediction equations of England's Health Survey). *Eur Respir J* **2004**, 23,456-463.
4. Langhammer A, Johnsen R, Gulsvik A, et al. Forzada valores espirométricos de referencia para adultos noruegos: la obstrucción bronquial en Nord-Trøndelag estudio (Forced spirometric baseline values for Norwegian adults: bronchial obstruction in Nord-Trøndelag study) *Eur Respir J* **2001**, 18, 770-779.
5. Borges RC, Barros JC, Oliveira FB, et al. Evaluation of pulmonary function and respiratory symptoms in pyrochlore mine workers. J Bras Pneumol. **2016** ,42,279-285.
6. Monterde S, Salvat I, Montull S, Fernández-Ballart J. Validación de la versión española del Fibromyalgia Impact Questionaire (Validation of the Spanish version of the Fibromyalgia Impact Questionnaire). Rev Esp Reumatol **2004**, 31, 507-13.
7. Burckhardt CS, Clark SR, Bennet RM: The Fibromyalgia Impact Questionnaire: development and validation. *J Rheumatol* **1991**, 18: 728–733.
8. Hoffmann TC, Glasziou PP, Milne R, et al. Better reporting of interventions: Template for Intervention Description and Replication (TIDieR) checklist and guide. *BMJ* **2014**;,348: g1687.
